# Bacteriophage-Mediated Control of Phytopathogenic Xanthomonads: A Promising Green Solution for the Future

**DOI:** 10.3390/microorganisms9051056

**Published:** 2021-05-13

**Authors:** Emilio Stefani, Aleksa Obradović, Katarina Gašić, Irem Altin, Ildikó K. Nagy, Tamás Kovács

**Affiliations:** 1 Départements of Life Sciences, University of Modena and Reggio Emilia, via Amendola 2, 42122 Reggio Emilia, Italy; emilio.stefani@unimore.it (E.S.); irem.altin@unimore.it (I.A.); 2 Plant Pathology Department, Faculty of Agriculture, University of Belgrade, Nemanjina 6, 11080 Belgrade-Zemun, Serbia; aleksao@agrif.bg.ac.rs; 3 Institute for Plant Protection and Environment, Teodora Drajzera 9, 11040 Belgrade, Serbia; gasickatarina@yahoo.com; 4 Nanophagetherapy Center, Enviroinvest Corp., Kertvaros u. 2, 7632 Pecs, Hungary; nagy.ildi.kata@gmail.com

**Keywords:** bacteriophages, bacteriophage therapy, biological control, *Xanthomonas* spp.

## Abstract

Xanthomonads, members of the family *Xanthomonadaceae*, are economically important plant pathogenic bacteria responsible for infections of over 400 plant species. Bacteriophage-based biopesticides can provide an environmentally friendly, effective solution to control these bacteria. Bacteriophage-based biocontrol has important advantages over chemical pesticides, and treatment with these biopesticides is a minor intervention into the microflora. However, bacteriophages’ agricultural application has limitations rooted in these viruses’ biological properties as active substances. These disadvantageous features, together with the complicated registration process of bacteriophage-based biopesticides, means that there are few products available on the market. This review summarizes our knowledge of the *Xanthomonas*-host plant and bacteriophage-host bacterium interaction’s possible influence on bacteriophage-based biocontrol strategies and provides examples of greenhouse and field trials and products readily available in the EU and the USA. It also details the most important advantages and limitations of the agricultural application of bacteriophages. This paper also investigates the legal background and industrial property right issues of bacteriophage-based biopesticides. When appropriately applied, bacteriophages can provide a promising tool against xanthomonads, a possibility that is untapped. Information presented in this review aims to explore the potential of bacteriophage-based biopesticides in the control of xanthomonads in the future.

## 1. Introduction

Plant diseases in pre- and post-harvest frequently account for 20% or more product losses, both in emerging countries as well as in developed areas [1]. Although less numerous than fungal diseases, bacterial diseases are often difficult to manage, due to their frequent polycyclic nature and the lack of systemic antibacterial substances [1]. Copper compounds and antibiotics are the only antibacterial choices to control phytopathogenic bacteria that are readily available in a large part of the world [2,3]. Copper presents several risks and unexpected consequences in agricultural systems and for the environment, e.g., phytotoxicity, negative effects on pollinating insects and other beneficial organisms, bioaccumulation in soil and surface water and reduction of microbial biodiversity [4,5,6]. Antibiotics, such as mainly streptomycin, kasugamycin and tetracyclines, as active substances in agriculture may also pose unacceptable risks when used as pesticides [3]. Indeed, although they do not accumulate or cause adverse effects on plants, they may incite the development of resistant traits in bacterial populations, including in the target pathogen(s), and transfer them to bacteria of clinical interest [7]. The urgent need to tackle pathogen control in agricultural systems using a more sustainable approach has directed research towards different strategies, among them the development and implementation of microbial biocontrol agents and bacteriophages [8,9]. In this review, we present the available knowledge on the use of bacteriophages in the management of xanthomonads, the largest group of phytopathogenic bacteria that are often the causal agents of devastating diseases in important crops. This review presents current knowledge on xanthomonads, bacteriophages, host-microbe interaction and ecology interactions. This information, -together with the description of results of relevant laboratory, greenhouse and field trials- supports the understanding of factors influencing the effectivity of bacteriophage-based biopesticides in the fields.

### 1.1. Xanthomonads

Xanthomonads are Gram negative bacteria belonging to the family of *Xanthomonadaceae*. Within this family *Xanthomonas* emerges as one of the most important genera in phytobacteriology, for it comprises around forty bacterial species pathogenic to over 400 plant species [10]. In turn, several *Xanthomonas* species are further taxonomically classified into different subspecies and pathovars, thus confirming a particular adaptation to plants. Such phytopathological adaptation is due to the expression of virulence factors [11,12]. Most *Xanthomonas* sp. strains are characterized by their production of xanthomonadin, a yellow pigment that represents the most useful diagnostic feature used for their identification [13], although a few pathovars are reported that do not produce such pigment, e.g.,: *X. axonopodis* pv. *manihotis*, *X. campestris* pv. *mangiferaindicae* and *X. campestris* pv. *viticola* [14,15]. Over the past 25 years, *Xanthomonas* species have undergone thorough changes in nomenclature based on phenotypic and conventional molecular techniques and, more recently, whole-genome sequencing (WGS) [16,17]. Indeed, evolutionary dynamics renders *Xanthomonas* species as rapidly evolving microbes and they are particularly successful as plant pathogens [14,18].

Several devastating plant diseases are caused by xanthomonads, for example *X. oryzae* pv. *oryzae* is the causal agent of bacterial blight, the most serious disease of rice. Together with pv. *oryzicola*, the causal agent of bacterial leaf streak, both pathogens frequently represent a limiting factor constraining rice production in tropical and subtropical regions [19]. Both pathogens exhibit large genetic variation among isolates, thus accounting for a high genetic plasticity [12].

The bacterial canker of citrus, incited by *X. citri* subsp. *citri* affects all commercial varieties of citrus [20]. Two other major crops are affected by xanthomonads: bananas (all types), affected by bacterial wilt caused by *X. vasicola* pv. *musacearum* and cassava, affected by bacterial wilt caused by *X. phaseoli* pv. *manihotis* [21]. International trade and climate change appear fundamental to support dissemination of xanthomonads worldwide and their adaptation and establishment in new areas, as several recent findings confirm [22,23,24].

### 1.2. Biological Control of Xanthomonads

Biological control of plant pathogenic bacteria may be implemented in several ways, for example (1) using microbial antagonists producing specific substances, such as bacteriocins (antibiosis), (2) using beneficial bacteria to efficiently compete for nutritional resources in planta [25], or (3) applying microbes that produce anti-Quorum Sensing factors [26], or (4) act as hyperparasites [27]. Emerging biocontrol strategies for plant pathogens, and for xanthomonads in particular, increasingly rely on the use of selected microbial biocontrol agents, or microbiome engineering [28,29]. Several microorganisms can efficiently control xanthomonads, both in vitro and in vivo, with some also showing plant growth promoting traits [30]. Specifically, bacterial species belonging to the genera *Pseudomonas* and *Bacillus* are reported to be effective against several *Xanthomonas* spp. A large number of papers describe satisfactory results on the biocontrol of *X. citri* pv. *citri*, *X. campestris* pv. *campestris* and *X. vesicatoria* [28], but most described results were obtained in vitro or in a controlled environment. Conversely, reproducibility of such published results in agricultural systems is not as good as expected, possibly due to the differences in agricultural context and the cropping systems. Nonetheless, a few commercial products based on microbial biocontrol agents that have satisfactory antibacterial activity are readily available on the market. For instance, Serenade^®^ and Serenade^®^ Max (Bayer Crop Science, Leverkusen, Germany) based on a selected strain of *Bacillus subtilis*, are indicated for the biological control of *X*. *arboricola* pv. *pruni*. Similarly, Double Nickel™ LC (Certis, Columbia, MD, USA) based on a strain of *Bacillus amyloliquefacies,* is indicated for the biological control of the tomato spot disease *(X. perforans)*.

### 1.3. Bacteriophages

Bacteriophages are viruses that specifically infect bacteria and have no direct negative effects on animals or plants. Bacteriophages are widely distributed on the Earth and are measurable components of the natural microflora [31]. In agricultural environments there are multiple sources of bacteriophages, such as healthy and diseased plant organs, soil, surface water, sewage and sludge, particularly from processing plants [32]. Bacteriophages may have different life cycles in natural environments. This includes a lytic life cycle, where a bacteriophage infects its bacterial host cell and rapidly induces its breakdown and a lysogenic cycle, where they are able to integrate their injected DNA into the bacterial genome [33].

Together with research on bacteriophages as prospective biocontrol agents (detailed in the Section 3), a number of studies were devoted to elucidating bacterial taxonomy. Bacteriophages have been used as tools to identify and characterize phytopathogenic bacteria [34]. Then, the use of specific bacteriophages appeared to be essential for population studies of phytopathogenic bacteria, in order to unravel key epidemiological factors. This supported the successful use of phages in controlling bacterial diseases [35].

Recent publications on isolation and characterization of bacteriophages against xanthomonads are summarized in Table 1.

## 2. *Xanthomonas*-Host Plant and Bacteriophage-Host Bacterium Interactions and Their Possible Influence on Bacteriophage-Based Biocontrol Strategies

No species is an island, as each individual organism is constantly in contact with others [50]. Here we discuss bacteriophage—host bacterium interactions and the factors that influence the possible outcomes of bacterial infection of the host plant. The presented data is helpful when identifying the non-satisfactory efficacy of bacteriophage-based pesticides when applied on the field and maybe useful when designing integrated plant management (e.g., with the involvement of other biopesticides). We provide possible solutions and explain why bacteriophage products may have distinct efficacies when applied on different fields. We will also analyze the applicable *Xanthomonas*-plant interactions from the point of view of biocontrol and the relevant bacteriophage-bacterium interactions. Finally, we will investigate the mechanisms of bacteriophage resistance of bacteria.

### 2.1. Xanthomonas-Host Plant Interactions

Bacteriophage-based biocontrol treatments of xanthomonads intend to interfere with a plant-pathogenic *Xanthomonas* spp. system. This subsection contains essential information on this system.

Xanthomonads live part of their life cycle outside the host plant as epiphytes in the lesions of fallen leaves or associated to plant debris in the soil [51]. They are an essential component of the soil microbiome, with 2–7% relative abundance in the bacterial community [52].

The infection cycle of *Xanthomonas* spp. starts with an epiphytic phase followed by entering the host plant through natural openings (stomata, hydathodes) and wounds to start its internal colonization (endophytic phase) [53]. When introduced into the plant surface, xanthomonads use a variety of adhesion strategies to attach to the plant [54,55,56,57,58,59]. Plants have also evolved various defence mechanisms to protect themselves from pathogens [60]. They respond to pathogen associated molecular patterns (PAMPs) by activating PAMP-triggered immunity (PTI) or effector-triggered immunity (ETI) mediated by pathogen-specific receptors [61]. As a result, a systemic acquired resistance (SAR) status may be established, potentially increasing resistance to subsequent attacks in the entire plant [62,63,64].

A first key element of bacterial survival in the phyllosphere is the biofilm formation, creating a microenvironment that can protect bacteria against environmental stress conditions [58,65]. This is an important virulence factor of phytopathogenic *Xanthomonas* spp. [66,67]. A biofilm, in addition to the cells, is primarily made up of proteins, lipids and extracellular polysaccharides (EPS) [68,69]. The formation of a biofilm may provide resistance to host defence mechanisms and vascular bacteria attachment to xylem vessels, or contribute to bacterial epiphytic survival prior to colonisation of the plant intercellular space [70]. The gum operon, a massive transcriptional unit containing 12 enzyme coding genes (*gum*B-*gum*M), mediates xanthan gum biosynthesis [71]. A study revealed that biofilm production deficient mutants (particularly *gum*B and *gum*D) showed significantly lower leaf surface survival than wild type *X. citri* pv. *citri* and *X. axonopodis* pv. *manihotis* [72,73,74]. The study of many *Xanthomonas* spp. have shown that gum genes contribute to bacterial in planta growth, epiphytic survival and disease symptom formation [72,75,76,77,78].

The assembly and dispersal of biofilms are partly mediated by the Quorum-sensing (QS) signal molecule, or diffusible signal factor (DSF). DSF positively influences the disruption of biofilms [79].

One survival strategy of bacteria during unfavorable conditions is the formation of persister cells. Persisters are a small fraction (0.001%–0.1%, or up to 1% in biofilms) of cells in a metabolically inactive, dormant state that are resistant against a wide range of antibiotics [80]. *X. campestris* pv. *campestris* and *X. citri* subsp. *citri* can form persister cells under different stress conditions [81,82]. Importantly, bacteriophages can also infect persisters [83].

LPS, as major components of the bacterial outer membrane, protect the cell from harmful environments and are another surface-associated virulence factor in *Xanthomonas* spp. Importantly, LPS not only function as virulence factors but also induce plant defense responses, such as pathogenesis-related gene expression, cell wall thickening and oxidative burst [84,85]. Mutations in LPS gene clusters make bacteria more susceptible to adverse environmental conditions, which may result in a reduction in bacterial virulence, as shown for *X. campestris* pv. *campestris* [86,87,88].

*Xanthomonas* species have a plethora of potential mechanisms that aid bacterial fitness in diverse environments, including the six different extracellular protein secretion systems (referred to as type I–VI, or T1SS–T6SS) that export proteins via the bacterial multilayer cell envelope and, in some cases, into host target cells. The conserved structural components that characterize these secretion systems, as well as the characteristics of their substrates and the pathway that these substrates take during the export process, distinguish them. T6SS was recently discovered and is involved in at least 25% of all sequenced gram-negative bacterial genomes [89]. The *Hcp* and *Vgr*G proteins are essential components of T6SS that mimic the bacteriophage tail and needle complex, respectively [90]. Yang et al. [90] investigated the evolution of the T6SS in the *Xanthomonas* genus and assessed the relevance of the T6SS for virulence and in vitro motility in *X. phaseoli* pv. *manihotis* (Xpm), the causal agent of cassava bacterial blight. According to their phylogenetic analyses, the T6SS may have been obtained through a very ancient event of horizontal gene transfer (HGT) and preserved through evolution, implying their significance for host adaptation. They also showed that the T6SS of Xpm is functional and immensely contributes to motility and virulence.

Transcription activation-like effectors (TALEs) ensure plasticity in host adaptation for xanthomonads. TALEs have a repetitive domain governing the binding to promoters of host genes [91]. Novel TALEs could be created because this repetitive region is shared among TALEs, and recombination frequently occurs, as it was recently demonstrated in *X. oryzae* pv. *oryzae* [92]. These novel TALE encoding genes could be changed by HGT between bacteria, strengthening their host adaptation abilities [93].

### 2.2. Bacteriophage-Host Bacterium Interactions

When investigating ecological roles of bacteriophages in a *Xanthomonas* spp. population, it should be highlighted that the relationship between bacteriophages and their hosts could be both antagonistic and mutualistic, and the long-term survival of a bacteriophage population does not always require the lysis of its host. Therefore, bacteriophages are not predators, but either parasites or parasitoids of the host [94].

Bacteriophages can infect bacteria located in biofilms, albeit biofilms can provide a barrier for bacteriophage attacks compared to planktonic bacteria. This barrier is due to the physiological heterogeneity of the bacteria composing the biofilms, the secreted EPS, and the differential display of receptors on the host cell’ surface [94]. Bacteriophages can interact with biofilms of xanthomonads at several points. In a recent study Yoshikawa et al. [37] isolated the *X. citri* jumbo bacteriophage XacN1. They showed that the XacN1 genome encodes potential lytic enzymes such as cell wall hydrolases, C1 family peptidase, M23 family peptidases, lipase and chitinase. According to proteomic analysis, lipase, chitinase, and M23 family peptidases were discovered in the bacteriophage XacN1. They concluded that these enzymes may be necessary to disrupting the biofilm and initiating bacteriophage infection. Bacteriophages have evolved to counteract the biofilm barrier by using depolymerase enzymes on their capsids, and can also induce host lysis, allowing bacteriophages to degrade biofilm [95]. Furthermore, bacteriophage genomes carrying QS genes were detected in *Clostridium difficile* bacteriophage phiCDHM1 and three *Paenibacillus* bacteriophage genomes [96,97,98]. These genes can modify the biofilm disruption and other QS-mediated responses, including the decision on the lysogenic or lytic lifecycle of bacteriophages [97] or even the synthesis of virulence genes, as demonstrated in *X. campestris* [98].

Generally, the diversity of bacterial communities can support their adaptation to environmental circumstances [99]. If a community is more diverse, it is more stable as it can better adapt to the changing environment [100]. Prokaryotic viruses are essential in driving processes in microbial ecosystems [101,102]. In the absence of bacteriophages, one or several strains could become dominant in the niche, and other strains could be extinct, as was demonstrated in in vitro experiments [101,103]. Bacteriophages most likely infect the most abundant host strain, causing a decrease in its abundance (”kill the winner” principle). A consequence of this action will be a fluctuating selection, that increases diversity [103] and strengthens the community’s stability or adaptation ability. This may cause that bacteriophage-based pesticides can support the presence of xanthomonads on the fields when not applied carefully. Integrated disease management together with the application of carefully selected bacteriophages timed appropriately could be one solution.

The genome of lysed cells will be available for surrounding bacteria, providing them novel genetic information, which may also include pathogenicity-related genes, as recently shown in the case of the cherry pathogen *Pseudomonas syringae* pv. *morsprunorum* or in *X. albilinean*s [104,105]. Lytic bacteriophages increase the mutation rate in their host’s genome, even in genes not related to bacteriophage resistance/immunity [101]. This effect can drive both adaptation (short term) or evolution (long term) processes. These from point of biocontrol disadvantageous features of lytic bacteriophages (i.e., providing novel genetic material for surrounding bacteria, increasing the mutation rate in the host’s genome) could be managed by an integrated disease management. However, the mentioned drawbacks are less serious, for example, when lysogenic bacteriophages are applied in the fields. Lysogenic bacteriophages can protect bacteria carrying their genomes from superinfection (Superinfection: A second (delayed) bacteriophage infection of an already bacteriophage-infected bacterium) [106]. Horizontal gene transfer is one of the major factors (together with the mutations in avirulence genes) to evade host resistance [107,108,109]. The fact that 5–25% of the genome of *Xanthomonas* spp. originates from recombination events [110] highlights its importance in xanthomonads evolution and adaptation processes. Exchange of virulence factors between *Xanthomonas* spp. via HGT was observed in several cases [12].

The complexity of these HGT actions is demonstrated in the genome of a *X. anoxopodis* strain that contains a truncated bacteriophage genome carrying a gene resembling a plant protein that is induced during citrus blight disease [111].

As bacteriophages are often strain-specific, they can also act on the population level, influencing the population’s intraspecific composition. Consequently, lysogens can contribute to the colonization of new niches. When lysis is induced in a small portion of the lysogenic cells, from superinfection-protected bacterial populations, and the bacteria originally located in the niche to be colonized are not protected from the infection, the new population can use their lysogenic bacteriophages as a weapon against the indigenous cells (“kill the relatives” principle) [101]. On the contrary, native bacteria can protect themselves against colonization by sacrificing a part of the population and inducing their prophages’ lytic cycle [101]. Lysogenic bacteria can use their prophage weapon effectively, as observed in an in vitro experiment recently, where a lysogenic-lytic switch of bacteriophages to QS autoinducers strongly influenced the viral and bacterial abundance and diversity in soil communities [112].

There are examples of how lytic induction is carried out to optimize the multiplicity of infection (MOI). QS, encoded by either bacteria or bacteriophages, can influence this process [113,114]. Moreover, some bacteriophage genomes contain their own density monitoring equipment (the *arbitrium* system) and encode for small oligopeptides with which the bacteriophage density can be measured, as described in *Bacillus* bacteriophages [114,115]. Lysogeny is preferred when bacteriophages are abundant. Based on the described features of lysogenic and transducing bacteriophages, their field application may contribute to the adaptation and pathogenicity of xanthomonads, i.e., it may lead to unwanted effects. Therefore, the application of well-characterized, strictly lytic bacteriophages is advisable for bacteriophage-based biocontrol.

As bacteriophages and their hosts are not alone in the microflora, bacteriophages will meet their hosts with rare frequency when the living cell number of the host is low. Thus, one important consequence of the ”kill the winner” principle is that bacteriophages cannot reduce the living cell number of their hosts to zero in a community [116], a property which differs from most chemical antibacterial compounds.

We mentioned examples in this subsection, how bacteriophages (both lytic and lysogenic ones) can alter the strain and/or species abundancies in communities. The composition of *Xanthomonas* spp. population and/or the microbial community may be distinct in different fields which may be differentially influenced by the described effects of bacteriophages. In addition to the environmental factors, a result of this divergent influence may lead to a distinct outcome of bacteriophage-based biocontrol in fields, at least in several cases [117].

### 2.3. Bacteriophage Resistance in Bacteria

Bacteriophage-resistance mutations in bacteria usually come with a fitness cost, such as a decrease in virulence, which results in less disease severity. This is because many of the molecules taking part in bacteriophage attachment are also engaged in the virulence mechanism. As a result, mutations that lead to resistance commonly compromise virulence. There are a few examples of how mutations in bacteria surface structures lead to decreased virulence, such as mutation in the *X. campestris xan*A gene needed for xanthan and lipopolysaccharide synthesis, which significantly decreases the effectiveness of bacteriophage L7 adsorption [118].

Bacteriophage resistance in bacteria is one of the main concerns regarding the bacteriophage-based biocontrol strategies. A detailed understanding of bacterial resistance to bacteriophages and their interaction with plants play an important role in the design of bacteriophage-based biocontrol strategies of xanthomonads. To survive bacteriophage infections, bacteria have developed a wide range of protection strategies, including spontaneous mutations, restriction modification systems (R–M systems), and adaptive immunity through the CRISPR-Cas system [106]. The key mechanisms driving bacteriophage resistance are spontaneous mutations, which can grant bacteriophage resistance by altering the structure of bacterial surface components that function as bacteriophage receptors [119]. Furthermore, bacteria can acquire resistance through lysogenic bacteriophages that carry sequences in their genetic material which encode bacterial resistance or toxins and incorporated into the bacterial genome [120]. The mechanisms by which bacteriophages counteract the anti-bacteriophage systems of bacteria are poorly understood. Bacteriophages with the ability to acquire new receptor tropism can modify their receptor-binding protein, which means that when a host receptor changes to a mutated form, bacteriophages can recognize the altered receptor structure and thus overcome disturbance in receptors for bacteriophage adsorption [121]. Bacteriophages use various anti-restriction strategies to avoid the wide range of R–M systems. These modification genes encode a small protein that is transmitted to the cell with the viral genome, or it may instantly neutralize the host immune system by intervening with the formation or function of the CRISPR–Cas ribonucleoprotein [122]. Bacteriophages may use bacterial CRISPR–Cas systems to promote their own replication, allowing the phage to complete its lytic cycle [123]. When a bacterium develops resistance to a specific bacteriophage, it retains sensitivity to bacteriophages with various cell surface receptors. Bacteriophage-mediated selection can be used in disease management, for example, by combining various bacteriophages to broaden the host range and suppress resistance evolution [124] and/or reasonably combining bacteriophages and chemical control to establish synergies and decrease the likelihood of resistance evolution [125]. This implies that the application of a bacteriophage cocktail may be beneficial, even if bacteria quickly develop resistance, since resistant strains may be less fit, thus more treatable using another combined method.

## 3. Bacteriophage-Based Biocontrol of *Xanthomonas* spp.

### 3.1. Examples for Greenhouse and Field Trials

Shortly after their discovery, bacteriophages were evaluated for control of plant diseases, including those caused by *Xanthomonas* spp. Some of the first studies were conducted by Mallman and Hemstreet (1924) who isolated the “cabbage-rot organism” *X. campestris* pv. *campestris* from rotting cabbage and showed that the filtrate from the decomposed tissue could inhibit pathogen growth in vitro [126].

From the 1960s, a considerable number of studies explored the efficacy of phages for the control of bacterial spot of peach, caused by *X. arboricola* pv. *pruni* [127,128,129,130]. Civerolo and Keil [127] applied bacteriophages 1 h prior to inoculation by the pathogen and reduced bacterial spot severity on peach leaves to 22% compared to 58% for control plants under greenhouse conditions. Civerolo [128] found that preinoculation of peach seedling foliage with crude lysates of the bacteriophage mixtures resulted in 6–8% fewer infected leaves and a 17–31% reduction of disease compared to control plants. Application of premixed bacteriophage—pathogen suspension immediately before inoculation resulted in a 51–54% decrease of bacterial spot symptoms in peach seedlings. Zaccardelli et al., isolated eight bacteriophages active against *X. arboricola* pv. *pruni*, examined their host range and lytic ability, and selected a lytic bacteriophage strain with the broadest host range for disease control [129,130]. By weekly bacteriophage treatment they significantly reduced fruit spot incidence on peaches [130].

Significant achievements have been made in bacteriophage application for control of bacterial spot of tomato caused by *X. campestris* pv. *vesicatoria* in greenhouse and field conditions [131,132,133,134,135,136,137,138]. Flaherty et al. [131] used a mixture of host range mutant bacteriophages and effectively controlled tomato bacterial spot in greenhouse and field conditions. Moreover, bacteriophage application increased total weight of extra-large fruit comparing to nontreated control or plants treated with chemical bactericides. Balogh et al. [133] improved the efficacy of bacteriophage treatments in field and greenhouse experiments by using protective formulations that significantly increased bacteriophage longevity on the plant surface. Bacteriophage mixture formulated either with 0.5% pregelatinized corn flour, Casecrete NH-400 with 0.25% pregelatinized corn flour, or 0.75% powdered skim milk with 0.5% sucrose, provided significant disease control compared to untreated control. However, in greenhouse experiments skim milk gave the best results, while Casecrete performed best in the field [133].

In order to improve bacteriophage efficacy and provide consistent disease control, bacteriophages of *X. campestris* pv. *vesicatoria* have been studied as a part of integrated disease management practices [138]. Obradovic et al., tested various combinations of plant inducers and biological agents for control of tomato bacterial spot [139]. Acibenzolar-S-methyl applied in combination with bacteriophages formulated with skim milk and sucrose, reduced bacterial spot of tomato in a greenhouse [136] as well as in the field [135]. Recently, Abrahamian et al. [140] evaluated 19 different chemical agents, biological control agents, plant defense activators, and novel products for their ability to manage bacterial spot on tomato caused by *X. perforans*. They reported that combination of bacteriophages, cymoxanil, famoxadone and phosphoric acid, significantly improved the disease management compared to the copper-based standard treatment. All these studies led to bacteriophage treatment, integrated with other disease management practices (e.g., late blight), becoming a part of a standard integrated management program for tomato bacterial spot in Florida [138,139].

Gašić et al. [141] studied the efficacy of bacteriophage KФ1 in the control of pepper bacterial spot caused by *X. euvesicatoria*. They found that double bacteriophage application, before and after challenge inoculation, significantly reduced disease incidence when compared to untreated control. However, integrated application of bacteriophages 2 h before and copper hydroxide 24 h before inoculation was the most efficient treatment. The same bacteriophage strain was used as a part of integrated disease management and combined with other biocontrol agents, copper compounds, antibiotics and plant inducers to control pepper bacterial spot [142]. Bacteriophage combination with copper-hydroxide and acibenzolar-S-methyl was the most effective treatment reducing the disease severity by 96–98% compared to control [142].

Similar studies were performed to develop management strategies for efficient and sustainable control of leaf blight of onion, caused by *X. axonopodis* pv. *allii*. Lang et al. [143] reported that biweekly or weekly applications of bacteriophages reduced disease severity in the field by 26 to 50%: similar to results achieved by weekly applications of copper-mancozeb. Therefore, integrated application of bacteriophage mixtures with acibenzolar-S-methyl could be a promising strategy for managing *Xanthomonas* leaf blight of onion and contribute to reduced use of chemical bactericides [143].

Comprehensive research was done on bacteriophage-mediated control of Asiatic citrus canker caused by *X. axonopodis* pv. *citri*, and citrus bacterial spot *X. axonopodis* pv. *citrumelo* [144,145,146]. Bacteriophage treatment, without skim milk formulation, provided an average 59% reduction in citrus canker severity in greenhouse experiments. In nursery, bacteriophage treatment reduced disease, but was less effective than copper-mancozeb, while bacteriophage integration with copper-mancozeb resulted in equal or less control than copper-mancozeb application alone [145]. Similar results were obtained in the management of citrus bacterial spot, where bacteriophage treatment provided significant disease reduction on moderately sensitive Valencia oranges while it was ineffective on the highly susceptible grapefruit [145]. Ibrahim et al. [146] reported that successful control of Asiatic citrus canker in greenhouse and field can be obtained by combination of bacteriophage mixture formulated with skim milk-sucrose and acibenzolar-S-methyl.

Initial research of bacteriophage infecting *X. oryzae* pv. *oryzae*, the causal agent of bacterial blight of rice, was conducted by Kuo et al., who applied purified bacteriophages 1, 3, and 7 days before inoculation, and obtained 100%, 96% and 86% reductions of bacterial leaf blight, respectively [147]. Recently, Chae et al. [148] significantly reduced the occurrence of bacterial leaf blight to 18.1% compared to 87% in untreated control by treatment with skim milk formulated bacteriophages. Ogunyemi et al. [149] reported the bacteriophage X3 was more effective in disease severity reduction (83.1%) if sprayed before inoculation rather than after (28.9–73.9%) it. However, seed treatment with bacteriophages reduced disease by 95.4%.

Other results on using bacteriophages specific to *Xanthomonadaceae* in plant disease control includes reduction of incidence of bacterial blight of geraniums caused by *X. campestris* pv. *pelargonii* with foliar application of h-mutant bacteriophages [150]. Nagai et al. [151] found that a non-pathogenic *Xanthomonas* sp. strain mixed with bacteriophages effectively controlled black rot of broccoli caused by *X. campestris* pv. *campestris* in field trials. Orynbayev et al. (2020) studied effects of bacteriophage suspensions mixed with different UV-protectants in control of black rot caused by *X. campestris* pv. *campestris* on cabbage seedlings. In two-year greenhouse experiments, bacteriophage DB1 mixed with 0.75% skimmed milk showed an average efficacy of 71.1% in control of the disease, compared to 59.1% efficacy of Kocide 2000 treatment [152].

A list of recent studies regarding bacteriophage-based biocontrol of diseases caused by *Xanthomonas* spp. is presented in Table 2.

### 3.2. Advantages

Low efficacy of available control strategies against xanthomonads is a consequence of the high multiplication rate of pathogens, high rate of pathogen mutation or gene transfer and thus increased population variability, as well as lack of efficient chemical treatments. Due to the difficulties in controlling *Xanthomonas* spp. incited diseases using conventional methods, considerable efforts were made to develop biologically based strategies.

Unlike chemical products for the treatment of bacterial diseases, application of bacteriophages has several advantages [138]. They have no toxic effects on the environment, and do not target eukaryotic cells [153]. Bacteriophages are self-replicating, and multiply exponentially upon infecting host bacteria. They are highly host specific, without affecting non-target organisms. Although they may vary in the host preferences, there are bacteriophage strains with a very narrow host range, infecting only a particular bacterial species or strain. Consequently, bacteriophages can be used against specific bacterial species without affecting beneficial microbial communities in the environment [154]. Thus, application of bacteriophages could contribute to maintaining beneficial or desired bacterial populations by suppressing the targeted ones. Additionally, bacteriophages can be used to target copper- and antibiotic-resistant bacteria, thus eliminating resistant strains [140,155]. They are suitable for mixing with the majority of chemical or biological crop protection products without significant effects on their efficacy [141,142,143]. They can be used to protect fresh-cut vegetables and fruits from post-harvest bacterial diseases [129] as well as human pathogens [156]. Additional advantages of using bacteriophages in plant protection come from their natural abundance and that their population dynamics is exclusively connected to the host bacterium’s population changes. Thus, they fit well as biopesticides that can be used in organic production for eliminating bacterial pathogens.

### 3.3. Limitations

In the past, despite the promising early work, application of bacteriophages did not provide reliable and effective disease control [138]. Lack of knowledge about bacteriophage biology and epidemiology, complexity of different bacteria–bacteriophage systems and plant environment resulted in reduced interest in developing and implementing bacteriophage-based control strategies. As biological entities, bacteriophages are exposed to environmental factors (for example UV light and drought) that can limit their efficacy compared to synthetic bactericides’ performance. Therefore, in-depth understanding of the optimal conditions for bacteriophage replication ability in the target environment is required [138,157]. Although bacteriophages may be effective in one crop, they may not be as effective in other pathosystems [158]. Optimization of their biocontrol activity requires a series of field experiments, since in vitro and greenhouse trials may not fully reflect the harsh conditions of the real environment and different crops. To improve survival of bacteriophages on plant phyllo- and rhizosphere, under ambient conditions, many adjustments are needed.

Major concern comes from the detrimental effect of the sunlight UV spectrum. Direct sunlight significantly reduces bacteriophage survival in the plant phyllosphere. Although available research results demonstrate that various substances can be used to protect bacteriophages from UV-induced decay, better formulations are still needed. Additionally, formulations are required to address the negative effect of desiccation as well as limited bacteriophage persistance on the plant surface. Timing of bacteriophage applications is essential for facilitating a high bacteriophage population reaching the target bacterium successfully, and for maximizing bacteriophages’ efficacy in extremely harsh environments that affect their survival [159,160]. At least in the case of several commercial products (i.e., Erwiphage^®^ Plus, [161]) the producer suggest spraying the biopesticide after sunset.

Gill and Abedon [159] indicated that in the rhizosphere, the soil water content determines diffusion rate of bacteriophages through heterogeneous soil matrices. Due to low rates of bacteriophage diffusion and high rates of bacteriophage inactivation, low numbers of viable bacteriophages are available to lyse target bacteria. Biofilms can trap bacteriophages [162], soil clay particles can reversibly adsorb bacteriophages [163], and low soil pH can inactivate bacteriophages [164,165].

There is also a high probability of developing resistant bacterial strains to bacteriophages [166]. This may compromise the bacteriophage treatment efficiency, especially in case of using single strain or narrow host range bacteriophage strains. Application of bacteriophages in a cocktail could minimize this risk and offer a potential solution [167,168]. Using the strategy of bacteriophage application proposed by Jackson could also prevent occurrence of phage-resistant bacterial mutants [169]. In order to prevent emergence of resistant bacteria and maximize bacteriophage application effectiveness, the method of application, timing, mixture of several bacteriophage strains, association with protective compounds and/or amendment with non-host avirulent bacteria as a carrier should all be optimized. Eventually, the production process of a bacteriophage-based treatment needs to be optimized for stability, purity and quality of bacteriophage.

### 3.4. Commercial Products

In the last two decades, several bacteriophage-based products have been registered for control of bacterial plant diseases. OmniLytics, Inc., Sant Lake City, UT, USA, was the first company to receive EPA registration to use bacteriophages in agriculture. Their first bacteriophage-based bactericide, AgriPhage, contains bacteriophages specific to *X. campestris* pv. *vesicatoria* and *Pseudomonas syringae* pv. *tomato* for control of bacterial spot and speck of tomato and pepper. In addition, this company recently developed new bacteriophage-based biopesticides for control of tomato bacterial canker, fire blight of apple and pear, and citrus canker. Another company, Otsuka Pharmaceutical, developed XylPhi-PD, which contains bacteriophages infecting *Xylella fastidiosa, the* causal agent of Pierce disease of grape. This product was approved for use in the USA. In Europe, there are two companies with registered bacteriophage-based biopesticides: a Hungarian company Enviroinvest, developed Erwiphage^®^ Plus for the control of fire blight of apple, and the Scottish company APS Biocontrol Ltd., designed Biolyse^®^ PB, containing bacteriophages for the control of potato soft rots.

## 4. Legal Basis for the Registration of Bacteriophage-Based Pesticides

It is well known that microbial biocontrol agents (MBAs) are very promising bio-pesticides composed of living microorganisms. Although viruses should not be considered as such, the international regulatory framework groups viruses together with other true microorganisms, such as bacteria and fungi [170]. As for other pesticides, before registration and marketing, MBAs must undergo a thorough risk assessment to food safety and environmental fate [171]. The EU assessment procedure was first laid down in Directive 91/414/EEC, followed by regulation number 1107/2009 that repealed and succeeded the Directive. With its implementation, only 26% of registered active substances and Plant Protection Products (PPP) passed the review, compared with those under Directive 91/414/EEC [172]. Therefore, only one fourth of the active substances allowed in agriculture were not revoked, as a sign of increased attention to consumers’ and environmental safety. In the USA, the regulatory framework appears less complex, since only two authorities are involved, namely the Environmental Protection Agency (EPA) and the Food and Drug Administration (FDA). Conversely, in the EU at least four major authorities are involved: The Rapporteur Member State, where the applicant submits a dossier containing all relevant information on the MBA, the European Food Safety Agency (EFSA), providing a risk assessment and risk communication, the Directorate General for Health and Food Safety (DG SANTE) and the Standing Committee on Plants, Animals, Food and Feed (PAFF Committee). The member states are divided into three evaluation zones. At the end of the process, requiring 26–36 months, the MBA can be included in the list of approved active substances.

A global overview on patenting bacteriophages is given by Holtappels et al. [173], where they found a good correlation between published papers and patent documents. Granted patent claims are rather broad and cover application methodology. The growing publishing and patenting activity indicates that there is a thorough interest in bacteriophages [173]. Conversely, very few authorized biopesticides based on bacteriophages are available on the market. EFSA, in a published opinion, excluded bacteriophages from the Qualified Presumption of Safety (QPS) (^(^*^)^ The Qualified Presumption of Safety (QPS) list is a list of microorganisms intentionally introduced into the food and feed production chain. It was first established in 2007 and it is revised and updated annually by EFSA in the framework of market authorization.) list as: (1) the lowest level of taxonomic unit (order of *Caudovirales*) is considered too broad; (2) lack of thorough analysis at the genome level to distinguish transducing and non-transducing bacteriophages or whether they carry virulence determinants [174]. Then, safety issues raised in the EU are leading to relatively strict regulations and registration protocols before the products can be placed on the market. Finally, Svircev et al. [9], framing the future of bacteriophages in agriculture, correctly identified potential problems with bacteriophages as biocontrol agents. Apart from the possible development of phage resistance in the bacterial host, true obstacles for the possible registration of bacteriophages as MBAs are represented by the production of lysogens or pseudolysogens and, additionally, that bacteriophages could serve as vectors for mobile genetic elements, including antibiotic resistance genes, as reported by other authors [175].

## 5. Conclusions and Future Perspectives

Bacteriophages have been studied as potential biocontrol agents to manage plant diseases caused by bacteria. In this review we have focused of bacteriophages of xanthomonads. To date, most studies have been conducted in the laboratory or under controlled conditions, most commonly in greenhouses and laboratories (discussed in Section 3), indicating the need for more field trials to ensure true applicability in open conditions.

There are multiple technological and biological opportunities to apply bacteriophages as biological pesticides in agriculture (as described in the subsequent paragraphs). For example, the use of endolysin increases the efficacy of bacteriophage biocontrol. Endolysins are enzymes used by bacteriophages at the end of their replication cycle to degrade the peptidoglycan membrane of the bacterial host from within, thereby lysing the cell and releasing progeny virions. The use of endolysin alone is effective only against Gram-positive bacteria, but a solution should necessarily be found for its use against Gram-negative bacteria. Artilysin, the outer membrane-penetrating endolysin, may offer this solution [176]. There are some newly discovered Gram-negative lysins that have roles principally against the major antibiotic resistant organisms causing nosocomial infections [177].

In order to enhance the efficacy of a bacteriophage-based biopesticide, it is highly desirable to apply it in the frame of an integrated plant protection (IPP) strategy (results and examples are detailed in Section 3). IPP strategies involve avoiding the disease outbreak, forecasting, detecting, and monitoring infections, designing biological interventions based on standardized principles, and measuring the effects of such interventions [178]. When designing a successful bacteriophage-based biocontrol strategy, the plant-pathogen-bacteriophage interaction triangle should be considered [179]. For example, the titer and composition of the bacteriophage cocktail and the timing of treatment may require alterations based on targeting of an epiphytic pathogen compared to an endophytic pathogen. Additionally, the influence on bacteriophage cocktails of other chemical treatments on the same crop should be evaluated.

Another promising development is the absorption of bacteriophages into plants. Bacteriophage penetration into plants has been previously detected [180]. However, nowadays, bacteriophage-products exert their antimicrobial effects on the surface of the plants, therefore on bacteria during their epiphytic phase, and the proportion of incorporated bacteriophages is minimal [180].

Despite its promise, bacteriophage-mediated control of phytopathogenic bacteria has some limitations (detailed in Section 3.3).

One of the most significant limiting environmental factors with the use of bacteriophages in agriculture is the destructive effect of UV light. However, bacteriophages have been isolated against *Pseudomonas syringae* pv. *actinidiae* which can tolerate extended high UV-B doses [181]. In addition, a straightforward method for supporting the phages’ survival in the environment is the utilization of bacterial bacteriophage carrier systems, as published recently [182]. In these systems, a non-pathogenic epiphytic bacterium strain is applied that can serve as biological control agent, bacteriophage delivery system and enables bacteriophage multiplication increasing its population on the plant surface [182].

However, besides the opportunities, the use of bacteriophages as a biopesticide may encounter some legal obstacles in the EU (detailed in Section 4). The use of viruses in therapy, even those that are beneficial and useful, is extremely provocative and controversial. Considering consumers perception, which is very sensitive to any microbe-based product when applied to food: this appears the most critical hurdle to be overcome in order for bacteriophages to be widely used in agriculture and elsewhere [183]. Researchers, therefore, have an extremely important and urgent task to tackle, collaborating with national leaders and decision-makers on bacteriophages and their potential use as sustainable plant protection products. Moving forward, it is essential for researchers to use previous data [9,175,184] to expand this field of research, thereby strengthening support for the useful and safe applications of bacteriophages in plant protection among stakeholders and the public.

Bacteriophage-based pesticides could be positioned as premium products suitable for application when the use of chemical-based pesticides is not possible, for example the Erwiphage^®^ Plus in Hungary [161]. To fulfil this role, end-users (farmers) should be informed of the biological features of these active compounds, and features of storage and application of these products should be optimized to be more user-friendly. One important area that requires optimization is the prolongation of the presence of infective bacteriophages in the orchard.

In conclusion, there is great, but currently yet unexploited, potential for bacteriophage use to replace or reduce the number of agrochemicals, such as copper and antibiotics, used for the control of bacterial plant pathogens, and xanthomonads in particular. To achieve this, the properties of bacteriophage-based products should be improved, pipelines of necessary characterization of the composed bacteriophages should be standardized, clear guidelines of application of bacteriophage-based biopesticides needed to be provided, and the registration process should be simplified, especially in the EU.

## Figures and Tables

**Table 1:** List of recent publications on bacteriophages against *Xanthomonas* spp.

Host Bacteria, Disease Name and Host Plant	Description of Works Performed	Reference
*Xanthomonas fragariae* Angular leaf spot in strawberry	Isolation and whole genome sequence analysis of N4-like bacteriophage, named RiverRider, including its host range.	[36]
*Xanthomonas citri* Asian citrus canker	Isolation and genome sequence analysis of *Xanthomonas* virus XacN1, a novel jumbo myovirus, showing a wider host range then other *X. citri* bacteriophages.	[37]
*Xanthomonas oryzae* pv. *oryzae* Bacterial leaf blight of rice	Characterization of a novel phage Xoo-sp2, isolated from soil and its potential as a prophylatic agent in biocontrol of the disease.	[38]
	Isolation and complete genome sequence analysis of bacteriophage Xoo-sp13.	[39]
	Isolation and complete genome sequence analysis of a jumbo bacteriophage, Xoo-sp14.	[40]
	Isolation and analysis of the complete genome sequences of 10 OP2-like *X. oryzae* pv. *oryzae* bacteriophages	[41]
*Xanthomonas campestris* pv. *campestris*Black rot disease of kohlrabi	Evaluation of lytic activity of Xccφ1 bacteriophage in combination with 6-pentyl-α-pyrone (a secondary metabolite produced by *Trichoderma atroviride* P1) and the mineral hydroxyapatite for the prevention and eradication of bacterial biofilms.	[42]
	Isolation and characterization of specific bacteriophage (Xccφ1) able to control disease, and investigation of *X. campestris* pv. *campestris* and Xccφ1, applied singly or combined, on plant metabolome.	[43]
*Xanthomonas campestris* pv. *Campestris* Black rot of crucifers	Isolation of phage infecting *X. campestris* pv. *campestris* and characterization of the bacteriophage Xcc9SH3.	[44]
*Xanthomonas campestris* pv. *Campestris* Black rot of caulifower	Isolation and morphological, molecular and phylogenetic characterization of *X. campestris* pv. *campestris* specific bacteriophage named “Xanthomonas virus XC 2”	[45]
*Xanthomonas arboricola* pv. *Juglandis* Walnut blight	Isolation of 24 phages from soil and infected walnut aerial tissues. Two polyvalent bacteriophages, were characterized by their morphological, physiological and genomic analyses.	[46]
	Isolation and complete genome analysis of three bacteriophages, f20-Xaj, f29-Xaj and f30-Xaj, specific to *X. arboricola* pv. *juglandis*	[47]
*Xanthomonasvesicatoria* Bacterial spot of pepper	Isolation and complete genome sequence of a filamentous bacteriophage XaF13 infecting *X. vesicatoria*	[48]
	Isolation and complete genome sequence of *X. vesicatoria* bacteriophage ΦXaF18	[49]

**Table 2:** List of recent studies of bacteriophage-based biocontrol for diseases caused by *Xanthomonas* spp.

Level	Host Bacteria, Disease Name and Host Plant	Description of Works Performed	Reference
Greenhouse and field trials against xanthomonads	*Xanthomonas euvesicatoria* Bacterial spot of pepper	Characterization and whole genome analysis of KΦ1 phage infecting *X. euvrsocatoria*. Single bacteriophage treatment as well its integarion with copper-hydrovide was evaluated in greenhouse conditions.	[141]
		Study of efficacy of biocontrol agents (bacteriophage KΦ1 and two strains of *Bacillus subtilis* AAac and QST 713), systemic acquired resistance inducer (acibenzolar-S-methyl), a commercial microbial fertilizer (Slavol), copper-based compounds (copper hydroxide and copper oxychloride) in combination with or without mancozeb, and antibiotics (streptomycin sulphate and kasugamycin), in control of the disease.	[142]
	*Xanthomonas citri* subsp. *Citri* Asiatic citrus canker	Efficacy of applications of formulated bacteriophages with skim milk and sucrose or nonformulated bacteriophages combined with acibenzolar-S-methyl compared to copper bactericides applications in control of the disease on leaves under greenhouse and field conditions.	[146]
	*Xanthomonas perforans* Bacterial spot of tomatoes	Evaluation of 19 different chemical agents, biological control agents, plant defense activators, and novel products in control of the disease on tomato seedlings and in the field.	[140]
	*Xanthomonas campestris* pv. *Campestris* Black rot of broccoli	Study of the potential of nonpathogenic *Xanthomonas* sp. strain 11-100-01 (npX) mixed with bacteriophage XcpSFC211 (pXS) in control of disease in greenhouse and field conditions.	[151]
	*Xanthomonas campestris* pv. *Campestris* Black rot of cabbage	Evaluation of the effect of UV light factor on persistence of the phage mixed with different UV-protectors in vitro and in planta on young cabbage plants in greenhouse conditions, and its efficacy in control of the disease.	[152]
Commercialized products against xanthomonads	*Xanthomonas campestris* pv. *Vesicatoria* bacterial spot of tomato and pepper	AgriPhage, contains bacteriophages specific to *X. campestris* pv. *vesicatoria* and *Pseudomonas syringae* pv. *tomato* for control of bacterial spot or speck of tomato and pepper.	Omnilytics, nd.

## Data Availability

Not applicable.
